# Enhancing electric vehicle battery lifespan: integrating active balancing and machine learning for precise RUL estimation

**DOI:** 10.1038/s41598-024-82778-w

**Published:** 2025-01-04

**Authors:** Yara A. Sultan, Abdelfattah A. Eladl, Mohamed A. Hassan, Samah A. Gamel

**Affiliations:** 1 Mechatronics Department, Faculty of Engineering, Horus University-Egypt, New Damietta, Egypt; 2 Electrical Engineering Department, Faculty of Engineering, Mansoura University, Mansoura, 35516 Egypt; 3 Electronics and Communication Engineering Dept. Faculty of Engineering, Horus University, New Damietta, Egypt

**Keywords:** Active balance, Lithium‐ion battery pack, Remaining useful life estimation, Machine learning, Engineering, Electrical and electronic engineering

## Abstract

Electric vehicles (EVs) rely heavily on lithium-ion battery packs as essential energy storage components. However, inconsistencies in cell characteristics and operating conditions can lead to imbalanced state of charge (SOC) levels, resulting in reduced capacity and accelerated degradation. This study presents an active cell balancing method optimized for both charging and discharging scenarios, aiming to equalize SOC across cells and improve overall pack performance. The proposed system includes two balancing strategies: a charging balance that redistributes excess charge from high-SOC cells to maximize capacity, and a discharging balance that addresses low-SOC cells to extend discharge duration. Experimental results confirm that this method effectively reduces SOC disparities, enhancing both charging and discharging capacities. Additionally, to accurately predict battery lifespan and remaining useful life (RUL), seven machine learning models are evaluated using R-squared (R^2^) and Mean Absolute Error (MAE) metrics. Among these, k-nearest Neighbors and Random Forest models deliver the highest accuracy, achieving R^2^ values of 0.996 and above with low MAE, demonstrating strong predictive capability. The integration of active balancing and RUL prediction enables a feedback loop where balanced SOC levels promote battery health, and RUL predictions inform optimal balancing strategies. This comprehensive approach advances EV battery management, enhancing lifespan and reliability through proactive balancing and predictive insights.

## Introduction

### Motivations

The rapid increase in global fossil fuel use for transportation has significant environmental implications, as combustion engines emit CO₂ and carbon residues, contributing notably to greenhouse gas emissions. Electric vehicles (EVs) offer a viable solution to reduce these emissions from traditional internal combustion engines, with substantial global uptake, particularly in countries like Norway, China, and the Netherlands, where government incentives and environmental policies have spurred high EV adoption rates^1–3^. Worldwide, EV sales have exceeded 5 million, reflecting a substantial increase in EV usage and the momentum of the EV market^4,5^. A crucial factor in EV success is the performance and durability of battery systems, which are vital for vehicle range, efficiency, and cost-effectiveness^6^.

Lithium-ion (Li-ion) batteries are the preferred choice for EVs due to their high power density, capacity, and low self-discharge, making them highly suitable for vehicle use^7,8^. However, they are sensitive to temperature changes, which can impair performance and accelerate degradation when outside the ideal range. To optimize Li-ion battery performance, battery management systems (BMSs) are employed in EVs^6^. BMSs handle functions such as charging control, thermal management, state-of-charge (SOC) and state-of-health (SOH) estimation, and cell balancing^9^. These systems play a central role in regulating charge and discharge, monitoring cell voltages, equalizing cell states, and identifying faults, thus enhancing battery performance, capacity, and lifespan^10^.

A primary function of the BMS is capacity balancing during charge and discharge cycles. Battery capacity imbalances may stem from internal variations in manufacturing or external conditions like temperature and depth of discharge, potentially reducing the battery’s lifespan^11^. Cell balancing ensures uniform SOC and voltage levels across cells, using either voltage-based or charge-based algorithms that dictate active or passive balancing methods^12^. Passive balancing, which dissipates excess energy as heat, is simple but limited to smaller battery arrays. Conversely, active balancing improves efficiency by redistributing charge from cells with higher capacity to lower-capacity cells, thus enhancing battery performance and longevity^13^.

Machine learning (ML) algorithms have emerged as effective tools for predicting the remaining useful life (RUL) of batteries by analyzing historical and environmental data^14^. By recognizing intricate patterns within operational data, ML models offer insights into battery health and degradation, enabling proactive maintenance and optimized charging strategies^15^. Extending the lifespan of EV batteries is crucial for the sustainability and broader adoption of electric transport, and recent research has focused on approaches such as active balancing and ML-based RUL prediction to achieve this goal^16^.

**Fig. 1:**
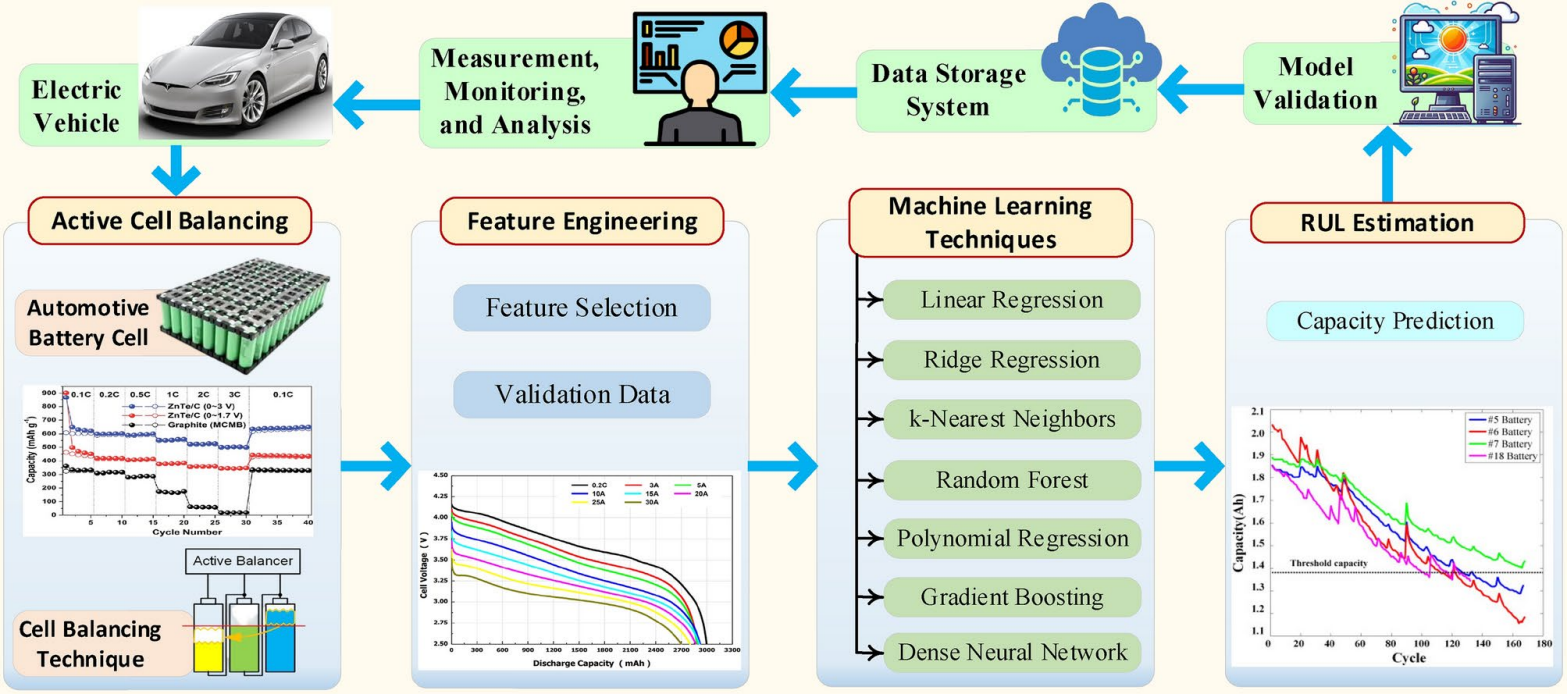
An overview of the comprehensive problem integration framework.

Combining active balancing with ML-based RUL estimation addresses both cell-level discrepancies and long-term health, as consistent SOC data enhances RUL model accuracy. Balanced cells contribute to better SOH across the battery pack, thus improving RUL predictions. ML algorithms that use balanced SOC data can more reliably estimate battery pack RUL, thus supporting longer EV battery lifespans and reliability. Figure 1 provides an overview of the framework, illustrating the integration between SOC estimation, RUL prediction, and the balancing strategy.

This study is motivated by the need to improve battery performance and lifespan, focusing on two key areas: advancing active cell balancing techniques and applying ML for RUL predictions. By refining methods to balance cell charge and discharge, we aim to ensure uniform energy distribution and sustain battery health. Additionally, by applying ML to analyze battery data, accurate RUL predictions can be achieved, facilitating proactive battery maintenance. This dual approach seeks to maximize battery potential and advance sustainability. This paper presents a novel active cell balancing control system that utilizes average SOC as a balancing parameter, incorporating an inductor for energy storage. Our goal is to explore the combined benefits of active cell balancing and ML-based techniques to enhance EV battery lifespan. By leveraging these approaches, we aim to establish a comprehensive framework that supports precise RUL estimation and effective battery management in EV applications.

### Related works

**Fig. 2:**
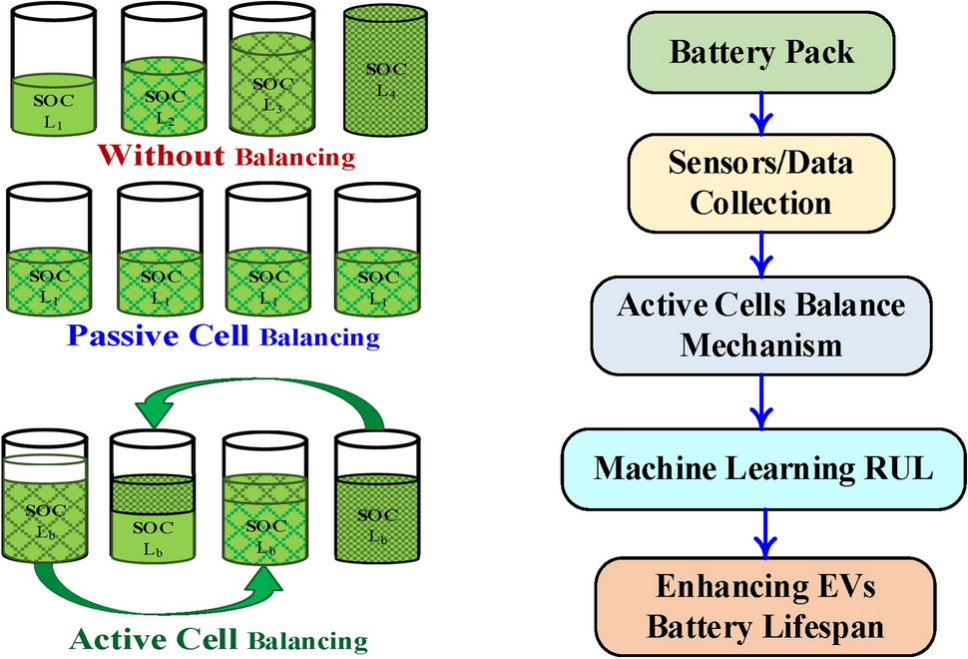
Method of cell balancing depending on battery’s level of charge and relation between cell balance and RUL.

Balancing processes are triggered by voltage fluctuations beyond a threshold, aiming for even charge distribution across cells. Passive and active balancing techniques are extensively analyzed in^17^, each with distinct pros and cons. Active balancing, though more complex and costly^18^, is particularly effective for large-scale battery systems by enhancing energy efficiency, capacity utilization, and battery lifespan. Despite the associated intricacies and expenses, these methods offer substantial benefits in terms of energy efficiency, capacity utilization, and the longevity of batteries. The discourse acknowledges the extensive research conducted on cell voltage balancing, which has explored a plethora of balancing methods. The distinction between passive and active cell balancing systems, as depicted in Fig. 2 and further elaborated in^19^, categorizes these systems into two primary categories. The passive system within the battery pack relies on balancing resistors to equalize cell voltages by dissipating excess charge from overcharged cells, whereas the active system employs a mechanism to transfer surplus charge from highly charged cells to those with lower charges, thereby conserving energy within the battery pack. Research into voltage balancing has investigated diverse methods, differentiating between passive and active systems^19^. Passive balancing dissipates excess charge as heat using resistors, which lowers efficiency^15–20^ and poses risks in lithium-based batteries^21^. Although simple and cost-effective, passive balancing lacks energy redistribution, leading to energy loss and prolonged balancing duration^8^. Innovations, such as MOSFET switches for variable resistance and ML optimizations^21^, still leave passive balancing best suited for low-power applications due to inherent inefficiencies^15^.

In contrast, active balancing aims to minimize SOC and state-of-health (SOH) disparities, which reduce uneven aging and degradation^16–31^. Using capacitive or inductive mechanisms, active balancing transfers excess charge to undercharged cells, enhancing uniform energy distribution^16–23^. While improving battery performance, active balancing introduces complex circuitry^24,25^. It encompasses various topologies, including capacitor-, inductor-, and transformer-based methods, each with trade-offs^16^. Capacitor-based balancing is economical and compact, yet slow^22^; transformer-based methods provide fast balancing but are costly and complex^23^. Inductor-based systems offer a compromise between speed and cost^24^, with advancements like linked inductors reducing component count^26^. Such methods, though intricate, are advantageous for high-power applications where efficiency and swift balancing are prioritized. These advanced circuits enhance the effectiveness of ML models in predicting remaining useful life (RUL), as balanced cells reduce data variability and improve model accuracy. Furthermore, utilizing a single switch per cell with unidirectional balancing streamlines the system while posing voltage stress on the center-cell switch^27^. Authors in^28^ offered a method to address prolonged balancing duration by integrating separate equalizers at multiple levels. Additionally^29^, introduced an any-cell-to-any-cell bidirectional inductor-based topology to enhance equalization current and efficiency. Despite their effectiveness in enhancing system efficiency and achieving faster cell balancing compared to passive techniques, these methods entail intricate circuitry, thereby increasing the overall system cost. Consequently, active cell balancing finds suitability in high-power applications, with different circuit designs categorized based on energy storage components, including capacitors, inductors/transformers, and electronic converters.

SOH in lithium-ion batteries is evaluated by comparing discharge capacity and resistance with initial values^30^, reflecting critical battery health indicators. Usage and conditions, such as high temperatures, accelerate capacity degradation through mechanisms like lithium loss and electrode material cracking^31^. Battery lifespan typically ends when SOH falls below a threshold, requiring precise estimation to optimize maintenance and control strategies^32^. Current SOH estimation models, often based on offline data, adjust parameters using filtering techniques, like the Kalman filter^33,34^. However, these models may not capture all degradation mechanisms, as they usually rely on uniform cell behavior, which may not account for real-world variability^35^.

ML methods for SOH and RUL estimation leverage real-time data to establish correlations without relying on predefined models. Techniques such as regression, neural networks, and hybrid models, which merge physics-based models with ML, provide adaptive and accurate predictions^36,37^. Despite the accuracy of these models, challenges arise from data variability, requiring preprocessing, feature engineering, and substantial computational resources for real-time predictions. Integration with battery BMS demands compatible infrastructure and extensive testing to ensure reliable operation across diverse conditions. Nonetheless, the potential for optimized battery management through proactive maintenance and reduced costs supports the longevity and sustainability of battery technology, especially in EVs.

**Fig. 3:**
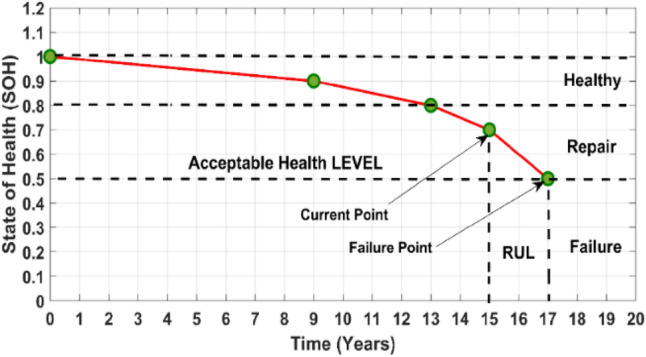
Remaining useful life prediction.

Figure 3 illustrates battery degradation over time, defining RUL as the interval from current status to potential failure. Precise RUL prediction allows proactive measures to prevent downtime and financial losses, underscoring the importance of accurate RUL estimation in maintaining operational efficiency and informed decision-making.

### Paper contributions and organization

This paper introduces an integrated approach for SOC balancing and RUL prediction to enhance battery performance and lifespan. The key contributions are as follows:

- An active balancing algorithm is proposed that reduces SOC imbalance with enhanced efficiency, minimizing energy losses and extending cell lifespan.
- Machine learning models are employed to provide accurate RUL estimation under various operating conditions, enabling robust battery life predictions.
- A comprehensive framework is presented that integrates SOC balancing with RUL prediction, optimizing both battery performance and longevity through real-time data adaptation.
- A comparative evaluation of the proposed approach is conducted against established SOC balancing and RUL prediction methods, demonstrating significant improvements in both accuracy and efficiency.

The paper follows a structured organization to comprehensively explore EV battery management and optimization. It begins with an introduction that outlines the motivations behind EV adoption and introduces the primary focus of the paper. Subsequently, the background section provides context by discussing the current state of EV adoption and emphasizing the significance of li-ion batteries. The paper then delves into the role of BMSs in maximizing battery performance, covering aspects such as charging control and cell balancing. Following this, it explores the importance of cell balancing techniques for charge and discharge management and investigates the potential of machine learning algorithms in battery management. Finally, the paper proposes a synergistic approach by integrating active cell balancing techniques with machine learning algorithms to optimize the lifespan of EV batteries, concluding with a summary of key findings and suggestions for future research directions.

## Methodology

### Battery pack imbalance indicator

Throughout operation, Li-ion battery packs experience charge and discharge cycles, resulting in inherent disparities in their stored energy levels. These differences materialize as deviations in terminal voltage, SOC, and internal resistance. Considering its encompassing nature, SOC, which encapsulates these parameters, was selected as the principal indicator for assessing cell imbalances within the battery pack in this manuscript. SOC denotes the readily available discharge capacity in relation to the battery’s nominal capacity and is commonly represented as a percentage, computed by dividing the remaining capacity by the full-charge capacity^38^.

1 $$SOC\left( t \right) = SOC_{int} - \frac{{\mathop \smallint \nolimits_{0}^{t} \eta I_{b} dt}}{{Q_{b} }}$$

where $SOC_{int}$ represents the initial state of charge, $t$ represents the accumulated time over which charge and discharge events have occurred, $I_{b}$ signifies the electrical current that is utilized in both the process of charging and discharging the battery, $Q_{b}$ defines the nominal energy capacity of a battery, measured in amp-hours and $\eta$ is the battery charging & discharging efficiency.

To assess battery pack imbalance and trigger cell balancing decisions, the standard deviation ${\upsigma }$ of individual cell SOC is leveraged as a key metric. A crucial imbalance is indicated when ${\upsigma }$ exceeds a predetermined threshold (${\upsigma }_{critical}$), which typically requires active balancing intervention. In contrast, ${\upsigma }$ values less than ${\upsigma }_{critical}$ signify satisfactory pack uniformity, thereby postponing the need for balancing. The average (μ) and standard deviation (σ) of SOC are computed as follows^39^:

2 $$\mu = \frac{1}{n}\mathop \sum \limits_{i = 1}^{n} SOC_{i}$$

3 $$\sigma = \sqrt {\frac{1}{n}\mathop \sum \limits_{i = 1}^{n} \left( {SOC_{i} - \mu } \right)^{2} }$$

where, $SOC_{{\varvec{i}}}$ defines the individual SOC of the $i^{th}$ battery cell (*i* = 1, 2,…, *n*), where *n* signifies the total number of cells in the battery pack.

### Equivalent circuit model for battery packs

While this paper focuses on SOC-based balancing methods, which are traditionally used in parallel-connected battery packs, the approach has been adapted to handle series-connected packs. For each cell in a series configuration, individual SOC values are monitored using real-time data from the BMS. When inconsistencies arise, the balancing strategy ensures that no single cell exceeds its cutoff voltage by transferring excess charge from higher SOC cells to lower SOC cells. This ensures both system safety and performance.

**Fig. 4:**
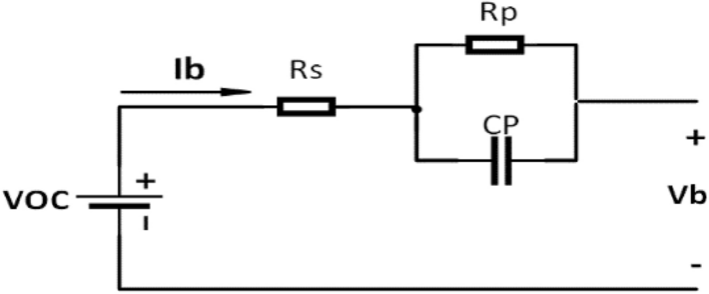
Equivalent circuit model of li-ion battery.

The typical way to depict the electrical characteristics of a battery is through an equivalent circuit. This circuit is built using basic electrical components like voltage sources, resistors, and capacitors. By combining these elements, we can approximate the intricate electrochemical reactions happening inside the battery and simulate its dynamic performance. Figure 4 illustrates the equivalent circuit model of the battery, consisting of three essential elements^40^:

- 1. **Equivalent Ohmic Internal Resistor (**${\varvec{R}}_{{\varvec{s}}}$**):** This component signifies the inherent resistance hindering current flow within the battery, attributed to factors such as electrolyte conductivity and electrode materials.
- 2. **Resistor–Capacitor Parallel Network (**${\varvec{C}}_{{\varvec{P}}} //{\varvec{R}}_{{\varvec{P}}}$**):** This segment replicates the battery’s transient response during charge and discharge cycles. It incorporates an equivalent polarization resistance (${\varvec{R}}_{{\varvec{P}}}$) to capture the voltage drop related to electrochemical processes within the battery during current flow. Additionally, it features an equivalent polarization capacitance (${\varvec{C}}_{{\varvec{P}}}$) to represent the storage and discharge of energy during transient voltage fluctuations.
- 3. **Open-Circuit Voltage (**${\varvec{V}}_{{{\varvec{OC}}}}$**):** This nonlinear function describes the battery’s voltage in the absence of current flow (open circuit), contingent upon the SoC of the battery.

The model regards the incoming current into the battery as the control input and the voltage detected at the terminals as the output. The ${\varvec{V}}_{{{\varvec{OC}}}}$ of the equivalent circuit model is represented as follows:

4 $$V_{OC} = V_{RS} + V_{{{\varvec{C}}_{{\varvec{P}}} //{\varvec{R}}_{{\varvec{P}}} }} + V_{b}$$

### Direct SOC estimation method independent of battery model

The direct estimation method of SOC, which operates independently of a battery model, hinges on easily measurable battery parameters that exhibit a robust correlation with SOC. These parameters typically encompass battery voltage, current, internal resistance, and impedance. Selection criteria for these parameters prioritize their ease of measurement during actual battery operation. Moreover, this approach offers notable advantages in terms of implementation simplicity, as it obviates the need for a complex and resource-intensive battery model^41^.

Direct measuring techniques, which do not rely on battery models, encompass ampere-hour integration, open circuit voltage, internal resistance, impedance spectroscopy, load voltage, and unique methodologies tailored for specialized applications. The ampere-hour integration method, often referred to as the coulomb measurement method or ampere-hour measurement method, calculates the SOC of a battery by summing the amount of electricity charged or discharged across each charge and discharge cycle. This method is characterized by its simplicity, straightforwardness, and minimal hardware and storage requirements for controllers.

### Active cell balancing circuit

In an equalizer utilizing an inductor as the energy storage component, the balancing circuit consists of a conventional Buck-Boost chopper circuit and a Buck chopper circuit. The equalizer regulates the balancing current by modifying the pulse-modulating signal (PWM) of the power switches to ensure that energy is transferred between the battery cells at a predetermined rate^42^.

**Fig. 5:**
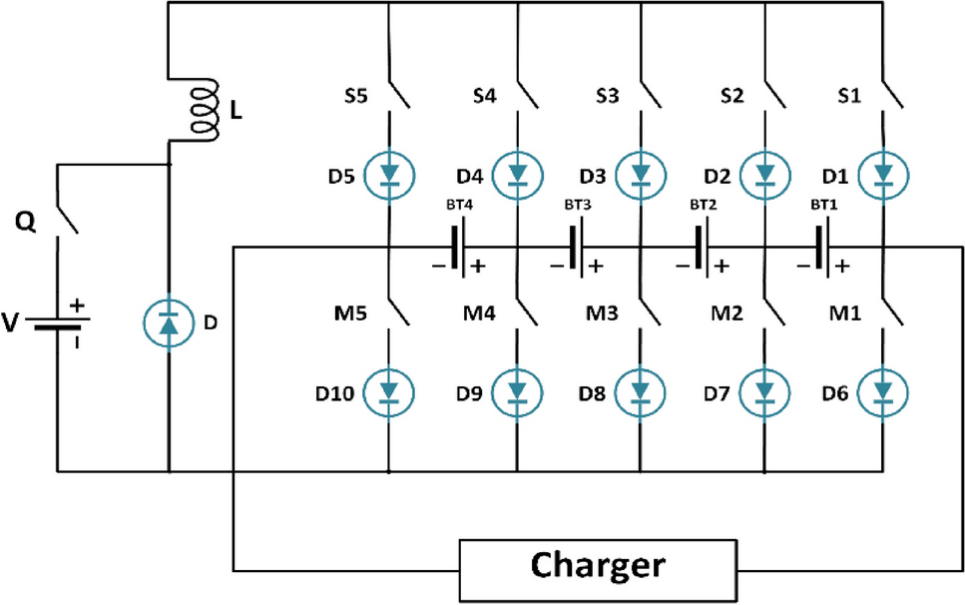
The battery circuit topology.

Figure 5 illustrates the battery balancing circuit topology designed for a four-cell series-connected battery pack. It incorporates an equalizer featuring two sets of power switches (M and S), an auxiliary power supply (V), an inductor (L), and a MOSFET power switch (Q). Each MOSFET switch includes series diodes to manage current direction and prevent short circuits. The energy transfer unit facilitates the regulation of energy flow from the high-energy cell to the entire battery pack using the auxiliary power supply V and inductor L. This transfer is orchestrated by adjusting the relevant power controls, enabling active charge and discharge balancing within the battery pack. Moreover, an external load is linked to the equalizer, contingent upon operational requirements. In Fig. 5, each battery cell connects to four power switches. By manipulating the activation states (on/off) of these switches in specific configurations, control over current direction is achieved. This manipulation allows for precise regulation of energy transfer from the battery. The active cell balancing strategy focuses on finely adjusting these power relays to attain the desired current and direction during battery charging and discharging.

The equalizer employs three control loops—Loop I, Loop II, and Loop III—to maintain the balance of the li-ion battery pack (LiB). During the charging process, Loop I and Loop II work together to balance the pack by transferring electrical energy from the cell with the highest SOC to the entire pack. This ensures that the energy is distributed evenly, preventing any single cell from becoming overcharged. Conversely, during discharge, Loop III ensures balance by transferring energy from an auxiliary power source to the cell with the lowest SOC. This helps maintain the discharge equilibrium across the pack, ensuring that no cell becomes overly depleted.

The operational details are as follows: when switches $M_{i}$ and $S_{i + 1}$ are closed, the current from battery cell $BT_{i}$ flows into inductor (*L*), transferring energy from $BT_{i }$ to L. Here, *i* represents the index of the battery cells (*i* = 1, 2, 3, 4), and Loop II refers to the battery energy feedback loop. The energy stored in inductor L is then transmitted to the entire battery pack when switches $S_{1}$ and $M_{5}$ are closed. Loop III acts as the charging loop for low-energy battery cells. By activating switches $Q$, $M_{i + 1}$, and $S_{i}$, a pathway is created for the auxiliary power supply’s energy to enter battery cell $BT_{i}$, which has a lower average SOC than the LiB pack. This ensures that even the cells with the lowest charge receive enough energy to stay balanced. In essence, Loop I and Loop II manage the charging equilibrium by facilitating the regulated transfer of energy from the cell with the highest SOC to the pack, while Loop III manages the discharging equilibrium by directing energy from the auxiliary power source to the cell with the lowest SOC, ensuring balanced discharge cycles.

The balancing approach is divided into two distinct operational periods of the LiB pack: charging and discharging. This methodology integrates specialized equilibrium techniques to ensure optimal performance and longevity of the battery pack.

- **Charging Balance:** This actively regulates cell voltages during the charging process to prevent overcharging and maintains a consistent SOC across all cells. This process ensures that each cell charges evenly, enhancing the overall efficiency and safety of the battery pack.
- **Discharging or Static Standing Balance:** Also known simply as discharging balance, this method addresses cellular imbalances that arise during periods of inactivity and discharge. By correcting these imbalances, the approach increases the pack’s overall efficiency and durability, ensuring that each cell discharges evenly and contributes to a longer battery lifespan.

### Active charge balancing strategy

**Fig. 6:**
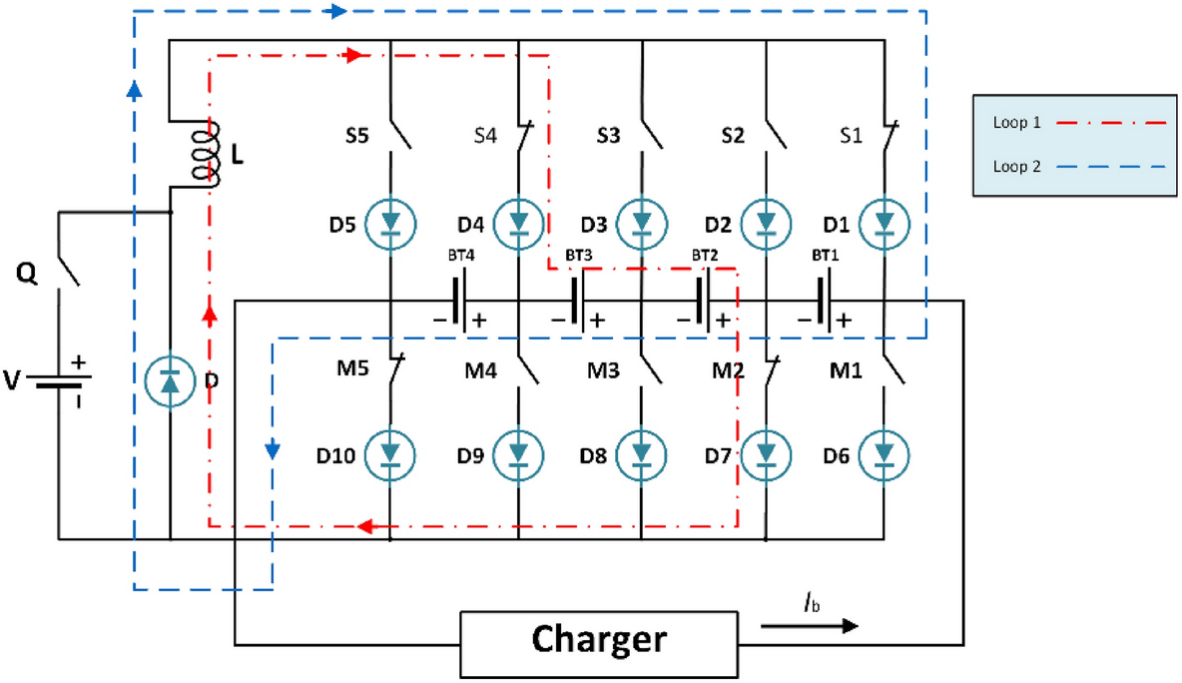
Balancing circuit during battery pack charging.

Figure 6 illustrates the active cell balancing circuit, while the external charger’s charging current is labeled as $I_{b}$. In a conventional Buck-Boost chopper circuit, the current flowing through the charged inductor is^43^

5 $$i_{L} \left( t \right) = - \frac{{V_{{BT_{i} }} - V_{d1} }}{{r_{1} }}e^{{ - \frac{{r_{1} }}{L}}} t + \frac{{V_{{BT_{i} }} - V_{d1} }}{{r_{1} }}$$

where, the variable $V_{{BT_{i} }}$ indicates the voltage of a battery cell $i$ that has been equalized, whereas ${\text{r}}_{1}$ represents the equivalent resistance of Loop I. The term $V_{d1}$ refers to the voltage drop across both the MOSFET and diode in the forward direction.

The inductor current attains its maximal value at the end of the MOSFET turn-on process.

6 $$i_{L}^{max} = - \frac{{V_{{BT_{i} }} - V_{d1} }}{{r_{1} }}e^{{ - \frac{{r_{1} }}{L}}} T_{on} + \frac{{V_{{BT_{i} }} - V_{d1} }}{{r_{1} }}$$

where $T_{on}$ represents the period of conduction for the MOSFET.

Given the fact that the MOSFET’s conduction time is determined by the switching period and duty cycle of the PWM, it is possible to rewrite the above equation in a simplified form that captures this dependency explicitly as follows

7 $${\text{i}}_{{\text{L}}}^{{{\text{max}}}} = - \frac{{V_{{BT_{i} }} - {\text{V}}_{{{\text{d}}1}} }}{{{\text{r}}_{1} }}{\text{e}}^{{ - \frac{{{\text{r}}_{1} {\text{D}}}}{{\text{L*f}}}}} {\text{T}}_{{{\text{on}}}} + \frac{{V_{{BT_{i} }} - {\text{V}}_{{{\text{d}}1}} }}{{{\text{r}}_{1} }}$$

where $f$ is the MOSFET switching frequency and $D$ is the PWM duty cycle.

The above equation demonstrates that with a constant duty ratio $D$, decreasing the switching frequency results in an increase in the conduction time of the MOSFET and a higher balancing current. The increased current necessitates the use of thick wire in the balancing circuit, which increases the weight and expense of the LiB pack and complicates the wire arrangement. However, when the switching frequency of the MOSFET is too high, the MOSFET loss becomes significant, and the high-frequency operating environment impacts the equalizer’s electromagnetic compatibility. In consideration of the MOSFET loss, balancing current, and balancing speed, the switching frequency of the MOSFET is established at 500 Hz.

When the switching frequency of the MOSFET is constant, a high duty ratio results in an extended conduction period for the MOSFET switches $M_{2}$ and $S_{4}$, as well as a significant balancing current. The extended conduction duration of the MOSFET switches $M_{2}$ and $S_{4}$ results in a decrease in the duration for which the MOSFET switches $M_{5}$ and $S_{1}$ are closed. During the discharge of the inductor, there is a possibility that the entire transfer of energy may not occur, leading to the saturation of the inductor.

Disregarding the equivalent resistance $r_{1}$ in the balancing loop, the average balancing current may be mathematically represented as

8 $$i_{C} = \frac{{V_{{BT_{i} }} - V_{d} }}{L}{*}\frac{D}{f}$$

Thus, the determination of the balancing inductance L is as follows:

9 $$L = \frac{{V_{{BT_{i} }} - V_{d} }}{{i_{C} }}{*}\frac{D}{f}$$

The current during the inductor discharge for four battery cells connected in series, is

10 $$i_{L} \left( t \right) = \left[ {i_{L}^{max} + \frac{{V_{A} + V_{d2} }}{{r_{2} }}} \right]e^{{ - \frac{{r_{1} }}{L}}} t - \frac{{V_{A} + V_{d2} }}{{r_{2} }}$$

where $V_{A}$ represents the overall voltage of the entire LiB pack, whereas $r_{2}$ is the equivalent resistance of the Loop Π. $V_{d2}$ represents the voltage drop that occurs in the forward direction across the MOSFET.

During the process of energy transfer, the inductor accumulates and stores energy throughout one switching cycle.

11 $$W_{1} = \mathop \smallint \limits_{0}^{{T_{c} }} Li_{L} \left( t \right)\frac{{di_{L} \left( t \right)}}{dt}tdt = \mathop \smallint \limits_{0}^{{T_{c} }} L\left( {\frac{{i_{L}^{max} }}{{T_{C} }}} \right)^{2} t^{2} dt = \frac{1}{3}LT_{c} \left( {i_{L}^{max} } \right)^{2}$$

where $T_{C}$ refers to the MOSFET’s switching cycle duration.

The electrical energy that needs to be balanced can be calculated as follows

12 $$Q_{d} = - Q_{b} SOC_{a}$$

where $SOC_{a}$ is the adjustment amount of soc.

The duration necessary to achieve a charging balance can be formulated as follows

13 $${\text{T}}_{{{\text{t}}1}} = {\text{nT}}_{{\text{c}}} = \frac{{3{\text{Q}}_{{\text{b}}} {\text{SOC}}_{{\text{a}}} }}{{{\text{L}}\left( {{\text{i}}_{{\text{L}}}^{{{\text{max}}}} } \right)^{2} }}$$

where $n$ is the number of switching cycles.

### Active discharging or static standing balancing strategy

**Fig. 7:**
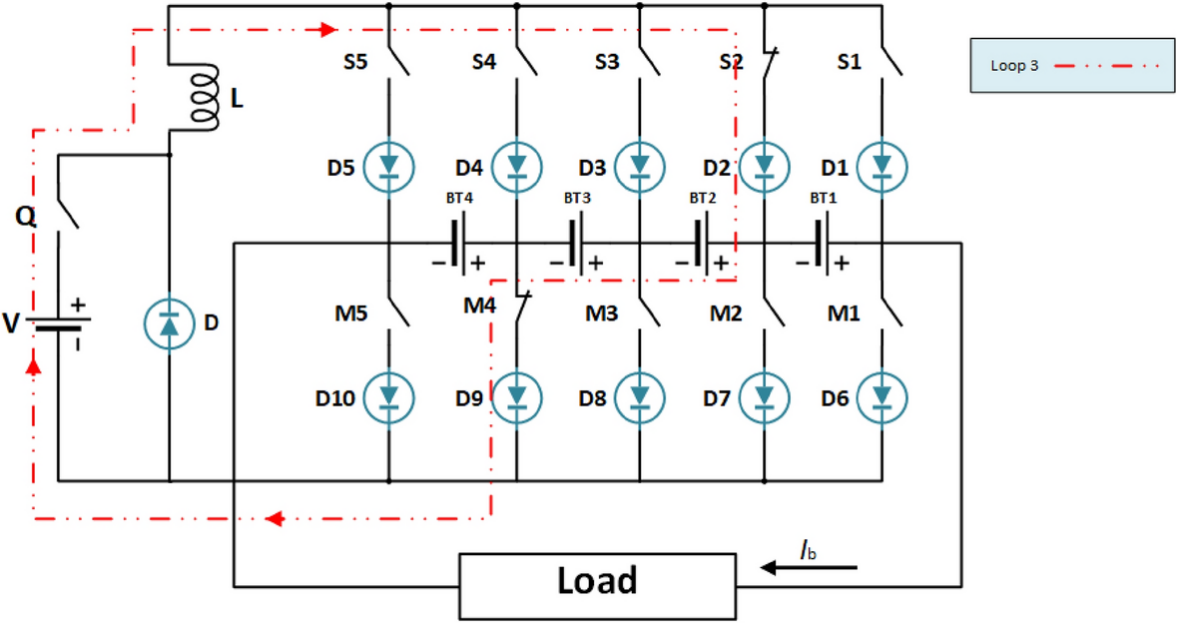
Balancing circuit during LiB pack discharging or static standing.

Under discharge or static standing conditions, the li-ion battery pack undergoes discharging balance to address cells with a SOC below the pack’s average. The balancing circuit associated with this process is illustrated in Fig. 7. This ensures that cells with lower SOC are brought up to the average level, enhancing the overall efficiency and durability of the battery pack by maintaining uniformity across all cells during discharge^44^.

During the discontinuous conduction mode (DCM) operation of the Buck chopper circuit, the average output voltage delivered by the balancing circuit can be expressed as follows

14 $$V_{0} = \frac{{D_{1} }}{{D_{1} + D_{2} }}{\text{E}}$$

where ${\text{E}}$ represents the DC voltage provided by the external power source that energizes the circuit, MOSFET Duty Ratio,$D_{1}$, signifies the fraction of the switching period during which the MOSFET remains turned on. Represents the ratio between the time the inductor spends discharging and the total period of operation. The expression for calculating this is given by the following relation:

15 $$D_{2} = - \frac{1}{2}\left( {D_{1} + \sqrt {D_{1}^{2} + 4\frac{2L}{{r_{2} T_{s} }}} } \right)$$

where the equivalent resistance for Loop III is represented by the variable $r_{2}$.

In terms of its average value, the balancing circuit’s output current can be calculated as follows:

16 $$i_{0} = \frac{{V_{0} }}{{r_{2} }} = \frac{{D_{1} }}{{D_{1} + D_{2} }}{*}\frac{{\text{V}}}{{r_{2} }}$$

The MOSFET’s switching frequency and duty ratio significantly affect the balancing current and speed. The above equations provide the following results:

17 $$i_{0} = \frac{{2{\text{E}}}}{{r_{2} \left( {1 + \sqrt {1 + \frac{8Lf}{{r_{2} D_{1}^{2} }}} } \right)}}$$

The above equation demonstrates an inverse relationship between the MOSFET’s switching frequency and the balancing current, assuming a constant duty ratio. Conversely, a fixed switching frequency exhibits a directly proportional relationship between the duty ratio and the balancing current. These observations necessitate critical design consideration, as both the MOSFET and the diode within the circuit incur losses during operation. To achieve a balance between speed and component efficiency, a MOSFET switching frequency of 500 Hz and a duty ratio of 50% were strategically chosen based on insights from relevant literature. For consistency, the duty ratio of switches $M_{4}$, $S_{2,}$ and $Q$ is also set to 50%. The amount of energy transferred per switching cycle can be expressed as

18 $$W_{2} = i_{0} T_{C} = \frac{{D_{1} }}{{D_{1} + D_{2} }}{*}\frac{{ET_{C} }}{{r_{2} }}$$

The equation for the balancing time required for a LiB pack, either during discharge or while stationary, is given by

19 $$T_{t2} = \frac{{Q_{b} SOC_{d} r_{2} }}{E}{*}\frac{{D_{1} + D_{2} }}{{D_{1} }}$$

### Results and simulation

**Fig. 8:**
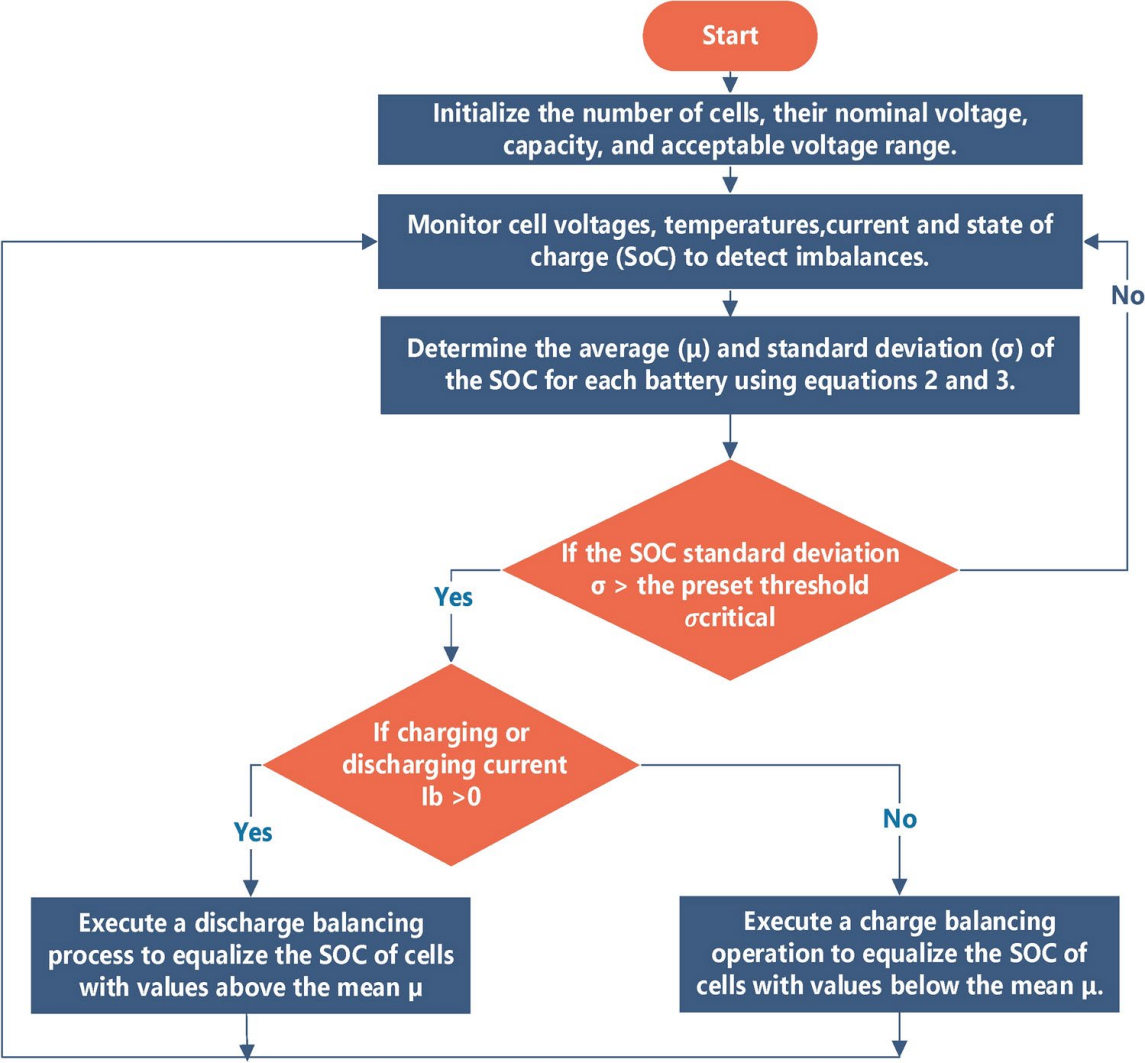
Cell balancing flowchart.

The manuscript investigates balancing techniques for batteries. It uses MATLAB/Simulink to simulate active balancing methods and to verify the effectiveness of the proposed method. A detailed illustration of the cell balancing algorithm for the LiB pack is presented in Fig. 8.

**Fig. 9:**
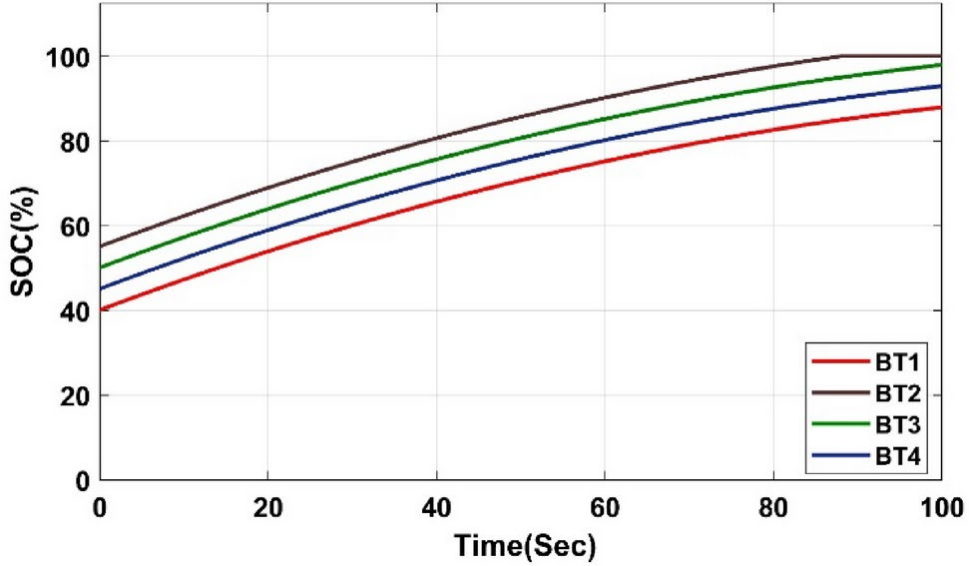
Evolution of SOC in four battery submodules under charging conditions.

The evolution of SOC in four battery submodules under charging conditions is shown in Fig. 9. The data reveals a significant improvement in battery SOC across all four batteries. BT1 jumped from 40 to 87%. BT2 achieves a full charge, going from 55 to 100%. Both BT3 and BT4 experience substantial gains, rising from 50 and 45% to impressive levels of 98% and 92% respectively. Table.1 quantitatively confirms the observations depicted in Fig. 9. It presents the SOC of each battery cell before and after active cell balancing, providing numerical evidence to support the significant improvement in SOC achieved by the balancing process. Several significant advantages are offered by this balancing technology over conventional methods, particularly in terms of efficiency, speed, and reliability:

- **Maximized Capacity Utilization:** This technology actively redistributes energy among cells with varying SOC levels, reducing energy loss as heat and charging lower SOC cells more effectively. This enhances overall battery capacity, optimizing performance and extending operational range.
- **Faster Balancing Speed:** The algorithm prioritizes cells with the largest SOC differences, enabling a faster and more efficient balancing process than standard methods, which improves energy equalization during both charging and discharging.
- **Improved Safety and Reliability:** Using an inductor-based system, energy is redistributed with minimal thermal stress, reducing heat dissipation and ensuring safer, stable operation compared to passive balancing.
- **Extended Battery Lifespan:** By maintaining lower SOC imbalances across cycles, this approach minimizes stress on cells, slowing degradation and supporting long-term battery health for applications requiring reliable energy storage.

**Table 1:** The SOC of battery cells before and after active balancing.

Battery	Start SOC (%)	End SOC (%)	Change
BT1	40	87	+ 47 (increased significantly)
BT2	55	100	+ 45 (reached full charge)
BT3	50	98	+ 48 (increased significantly)
BT4	45	92	+ 47 (increased significantly)

## Machine learning for accurate RUL estimation

### Dataset description

This dataset provides valuable information on the behavior of the batteries throughout the cycling process and can be utilized to develop predictive models for estimating the RUL of similar batteries. The dataset described originates from a study conducted by the Hawaii Natural Energy Institute, focusing on the analysis of 14 NMC-LCO 18,650 batteries with a nominal capacity of 2.8 Ah. These batteries underwent 1000 cycles at a temperature of 25 °C, utilizing a CC-CV (Constant Current-Constant Voltage) charge rate of C/2 and a discharge rate of 1.5C. The inputs to the RUL prediction models consist of key features that influence battery degradation, including:

- **SOC**: Captures the charge level, impacting battery health and capacity fade.
- **Temperature**: Reflects the thermal conditions, as high temperatures can accelerate degradation.
- **Voltage and Current**: Provide insights into operational load and charging/discharging patterns.

While, the model output is the RUL, expressed as the estimated number of cycles remaining until each cell reaches its end-of-life threshold. Derived features capturing voltage and current behavior throughout each cycle were extracted from the original dataset. Feature selection was conducted using a combination of domain expertise and statistical analysis to identify attributes most relevant to battery health and RUL prediction. Features with high correlation to battery degradation indicators, such as SOC range, voltage fluctuations, and charge/discharge cycles, were selected for their predictive value and influence on model accuracy. These features are potentially useful for predicting the RUL of the batteries. The dataset provides a summary of the 14 batteries used in the study, with the following variables^45^:

1. Cycle Index: Indicates the cycle number for each battery.
2. F1: Discharge Time (s): Duration of the discharge process in seconds.
3. F2: Time at 4.15 V (s): Time spent by the battery at a voltage of 4.15 V in seconds.
4. F3: Time Constant Current (s): Duration of the constant current phase in seconds.
5. F4: Decrement 3.6–3.4 V (s): Time taken for the voltage to decrease from 3.6 V to 3.4 V in seconds.
6. F5: Max. Voltage Discharge (V): Maximum voltage achieved during the discharge process.
7. F6: Min. Voltage Charge (V): Minimum voltage reached during the charging process.
8. F7: Charging Time (s): Duration of the charging process in seconds.
9. Total time (s): Overall time for the battery cycle in seconds.
10. RUL: Target variable representing the remaining useful life of the battery.

The dataset was divided into training and testing sets using an 70–30 split, where 70% of the data was used to train the model and 30% was reserved for testing. This split was selected to ensure sufficient data for model training while preserving a portion for evaluation. Random sampling was applied to maintain a balanced representation of various operating conditions, enhancing the generalizability of the model. This dataset offers valuable insights into battery behavior during cycling and can be leveraged to develop predictive models for estimating the RUL of similar batteries.

### Simulation and results

#### Data visualization and feature exploration

**Fig. 10:**
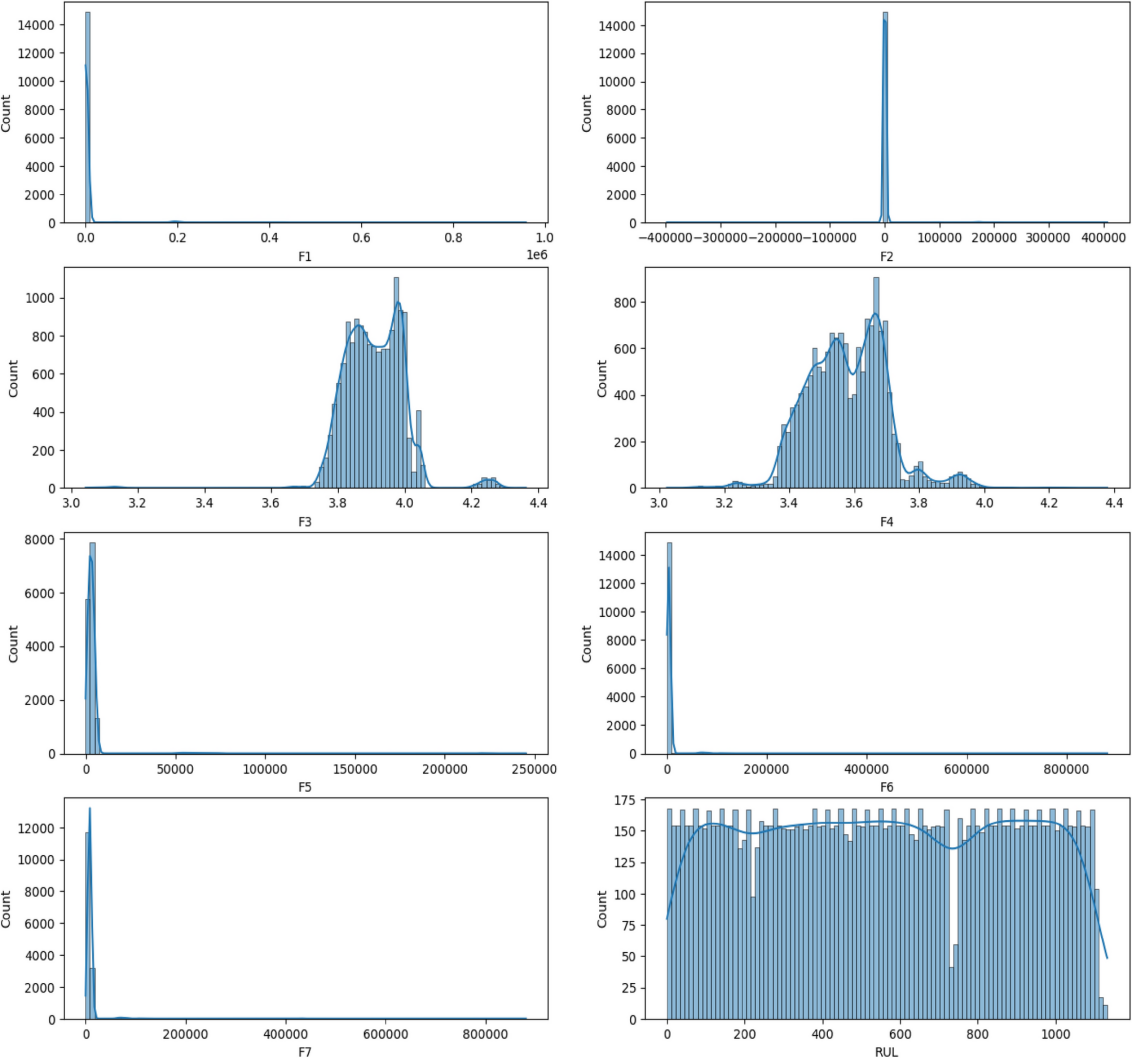
Distribution of features in dataset.

Before delving into data analysis or model building, a thorough understanding of the data’s characteristics is paramount. To achieve this, we employed data visualization techniques. Figure 10 presents the distribution of each feature within the dataset. These visualizations, often histograms or kernel density plots, depict the frequency with which specific data values occur. By analyzing the distribution, we can glean valuable insights into the data’s central tendencies (like mean or median), spread (variance or standard deviation), and potential skewness (lopsidedness towards one side). This information is crucial for informed decision-making during subsequent stages, such as feature selection or model selection. For instance, if a feature exhibits a highly skewed distribution, data transformation techniques might be necessary to ensure normality, which can improve the performance of certain machine learning algorithms.

**Fig. 11:**
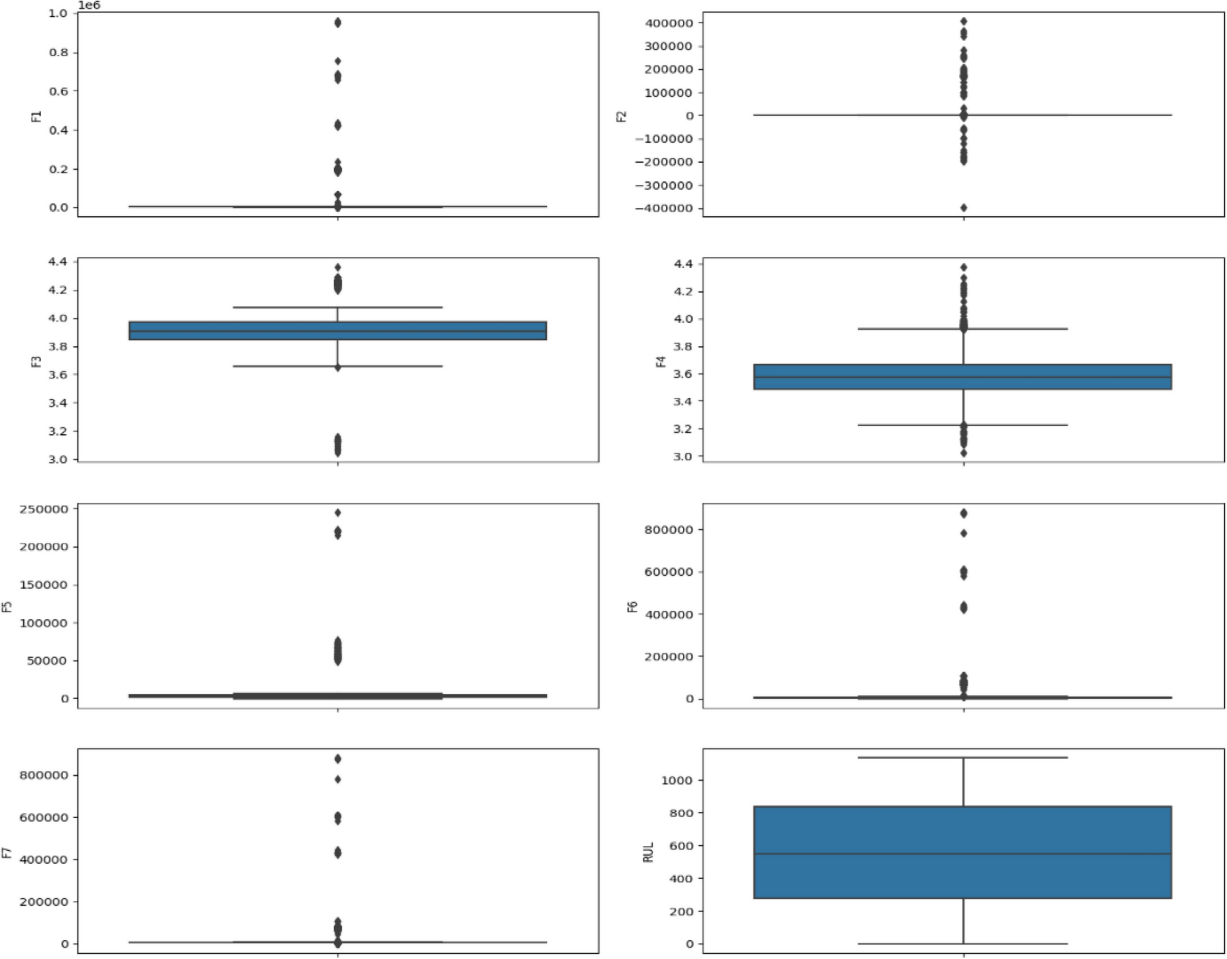
Boxplot of the dataset features.

Furthermore, we utilized exploratory data analysis (EDA) techniques to delve deeper into the data and identify potential anomalies. Figure 11 specifically showcases box plots, which are a visual representation of the distribution of a feature. Box plots reveal the median value (the center line), the interquartile range (IQR, the middle 50% of the data), and outliers (data points that fall outside a certain range). In our analysis, the box plots in Fig. 11 revealed the presence of outliers in features F1, F2, F5, F6, and F7. Outliers can sometimes represent genuine data points, such as extreme but valid observations. However, they can also indicate errors in data collection or processing.

#### Balancing accuracy and data integrity

To ensure the integrity of our analysis and mitigate the influence of potentially inaccurate values, we meticulously evaluated each outlier identified in features F1, F2, F5, F6, and F7. This evaluation involved differentiating between potential errors and valid extremes. Confirmed errors, arising from issues like data collection mistakes or typos during data entry, were carefully removed from the dataset. This ensures that the data accurately reflects the underlying population we are studying and prevents outliers from skewing the results of subsequent analyses or model training.

**Fig. 12:**
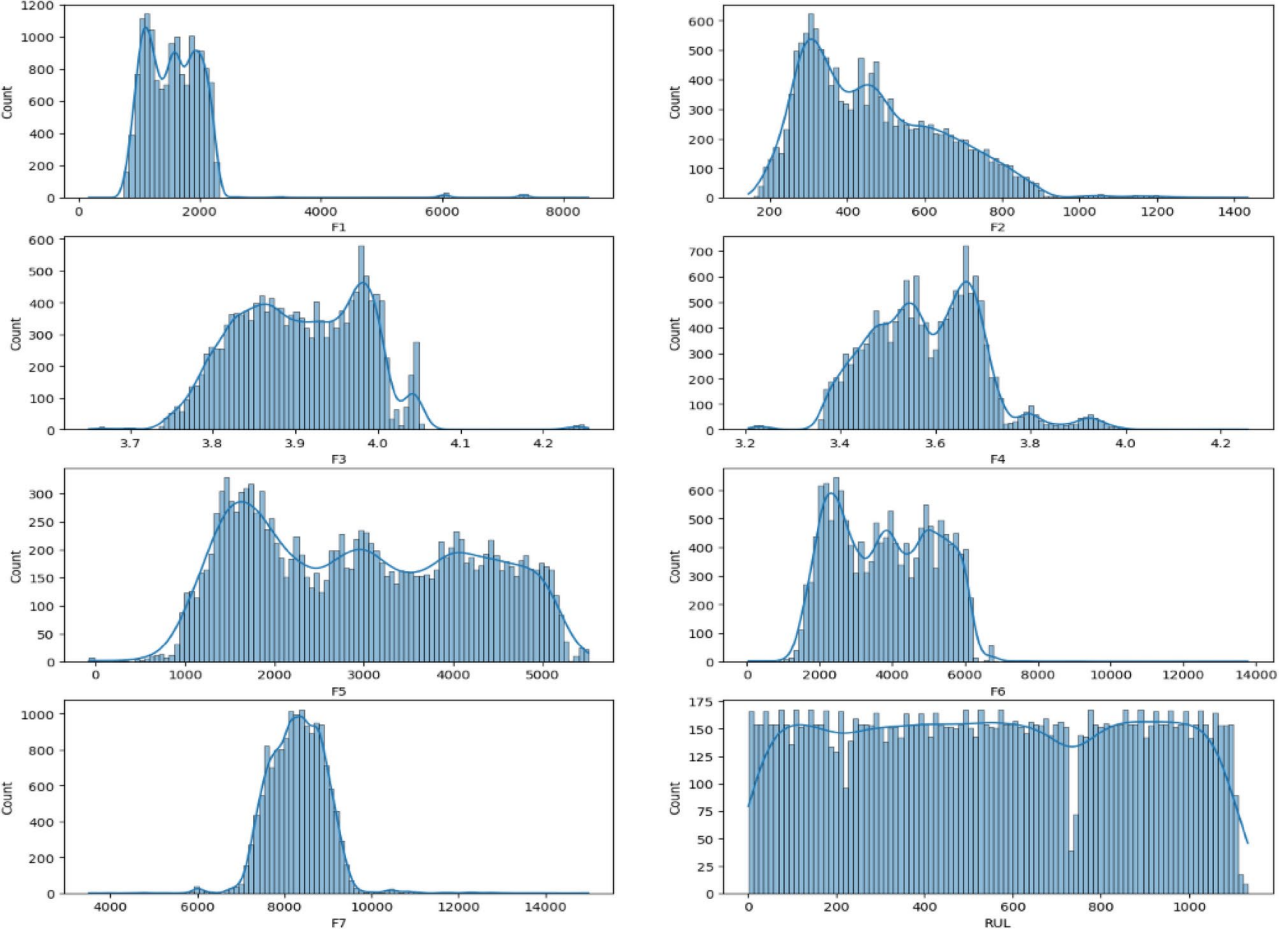
Distribution of features in the dataset before cleaning the data.

**Fig. 13:**
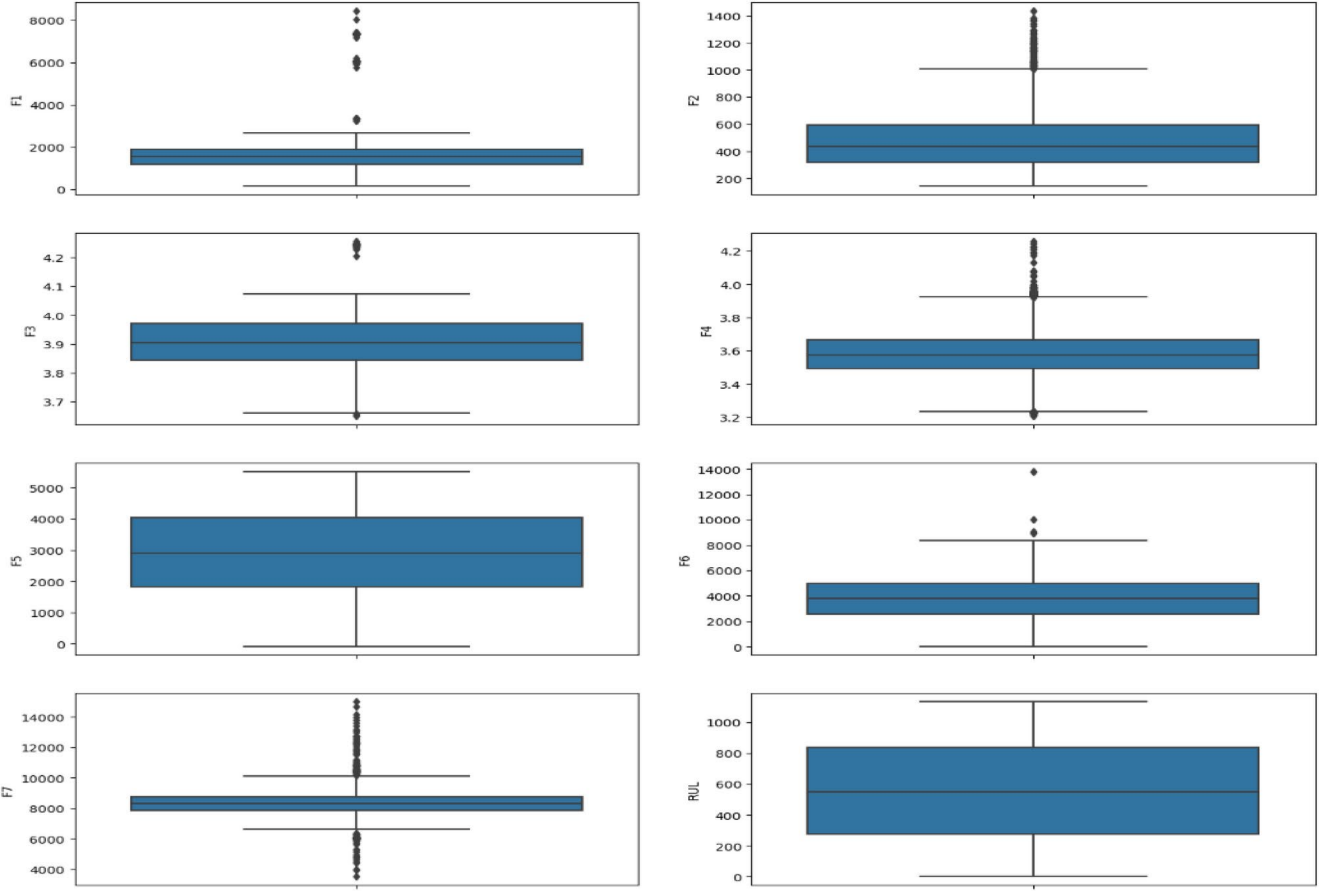
Boxplot of the dataset features after cleaning the data.

However, it’s important to strike a balance. Removing all outliers can be detrimental if they represent genuine, albeit extreme, data points. Therefore, each outlier was thoroughly examined and only verified errors were eliminated. This meticulous process ensures that the data remains representative of the population while mitigating the influence of potentially inaccurate values. This focus on data quality is crucial for building robust models that generalize well to unseen data. Outlier removal was conducted using systematic interquartile range (IQR) analysis to ensure data quality and model robustness. Observations falling beyond 1.5 times the IQR from the first and third quartiles were identified as outliers and excluded from the dataset. This objective method, widely accepted in machine learning applications, effectively filtered out extreme values likely caused by measurement errors or unusual operating conditions, which could otherwise skew predictions and model accuracy. By applying this approach, we maintained data consistency and ensured that the model’s performance reflects typical operating conditions rather than being artificially aligned with expected outcomes. The outcomes of this data cleaning step, including the removal of confirmed errors and the preservation of valid outliers, are illustrated in Figs. 12 and 13. These figures serve as a testament to the importance of data cleaning and its contribution to a more robust foundation for subsequent analysis and model training.

#### Data visualization and exploration efforts

As described previously, laid the groundwork for robust model development. Following the meticulous data cleaning process outlined in Figs. 11 and 12, we can be confident that the dataset is balanced and free from major inconsistencies. This ensures the data accurately reflects the underlying population of batteries under study and prevents outliers from skewing model results. The cleaned dataset serves as a solid foundation for our subsequent analyses.

**Fig. 14:**
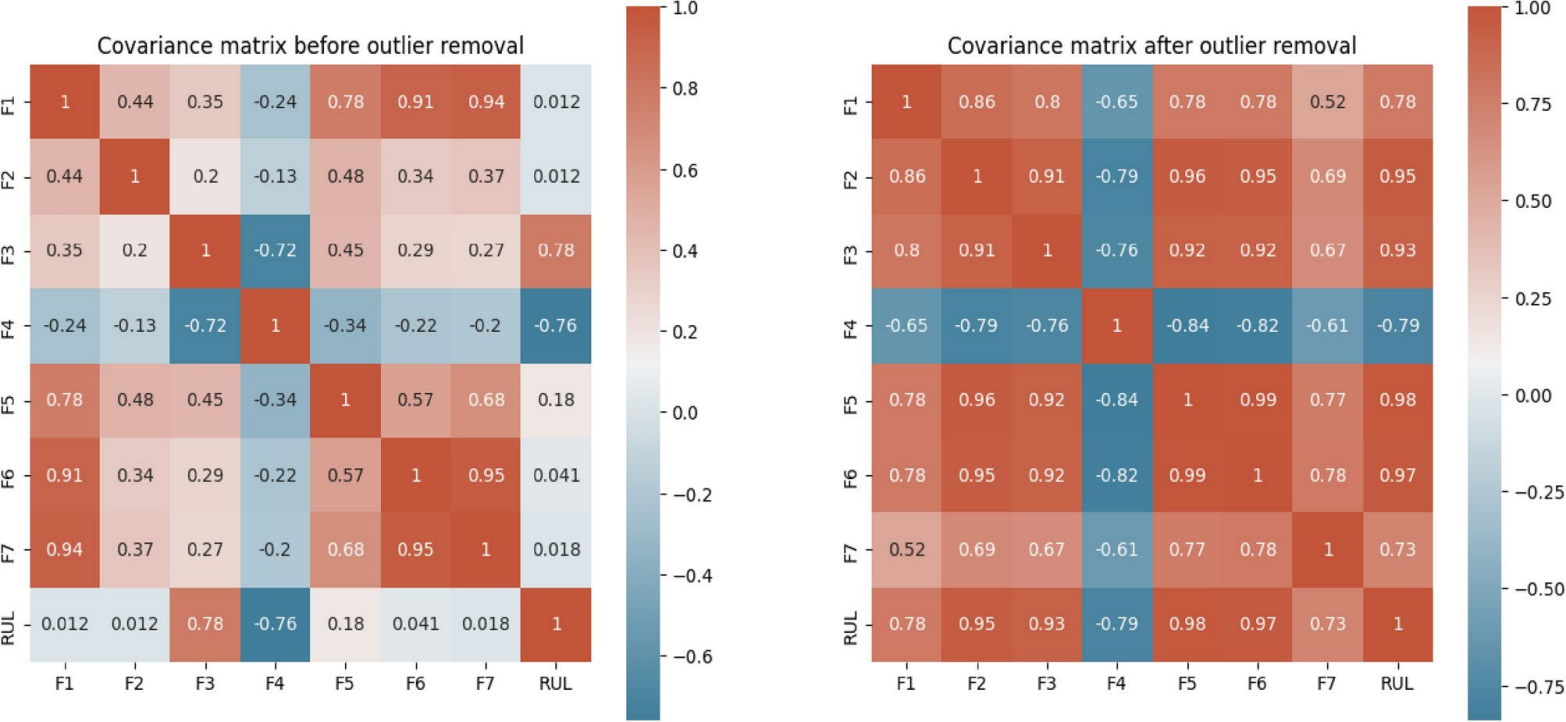
Heatmap between dataset features before and after cleaning the data.

Figure 14 takes our exploration a step further by delving into feature correlations. It presents the relationships between various features (F1-F7, likely voltage and current characteristics) extracted from the battery cycling data, both before and after data cleaning. Analyzing the trends in this graph allows us to discern how these features, potentially indicative of battery health, evolve throughout the cycling process represented by the cycle index. Understanding these trends is critical for predicting the RUL of the batteries, which is our ultimate goal.

By uncovering patterns and relationships between features and the cycle index, we can develop powerful predictive models. These models will learn to identify subtle changes in the battery’s behavior (represented by features) that signal impending degradation. This knowledge enables us to estimate the RUL with high accuracy. Ultimately, accurate RUL prediction empowers informed decision-making regarding battery maintenance and replacement strategies, maximizing battery lifespan and system reliability.

In our simulation, we experimented with various feature selection techniques. To ensure a fair and unbiased comparison of different machine learning algorithms for RUL prediction, implemented several measures were implemented:

- **Uniform Data Splitting:** Each algorithm was trained and tested on the same data split, using a consistent 70–30 division for training and testing. Cross-validation was also applied across all models to provide an additional layer of robustness and to minimize the influence of any single data split.
- **Standardized Dataset and Feature Selection**: All models were trained on the same dataset, using identical preprocessing steps and feature sets, to eliminate data-related biases.
- **Hyperparameter Optimization**: Each model underwent hyperparameter tuning using cross-validation. This approach optimized each algorithm’s performance, ensuring that results reflected the models’ capabilities rather than differences in parameter settings.
- **Uniform Evaluation Metrics**: We used consistent evaluation metrics, including Mean Absolute Error (MAE) and Root Mean Square Error (RMSE), to objectively assess the models’ predictive accuracy. This allows for a fair comparison of model performance across different algorithms.

The resulting best_features list contains seven sets of features, each identified as important for a specific prediction task. These sets highlight features consistently chosen by different selection methods, suggesting their potential significance for prediction. Further analysis will explore the relationships between these features and the target variable RUL to refine our understanding and potentially improve model performance.

**Fig. 15:**
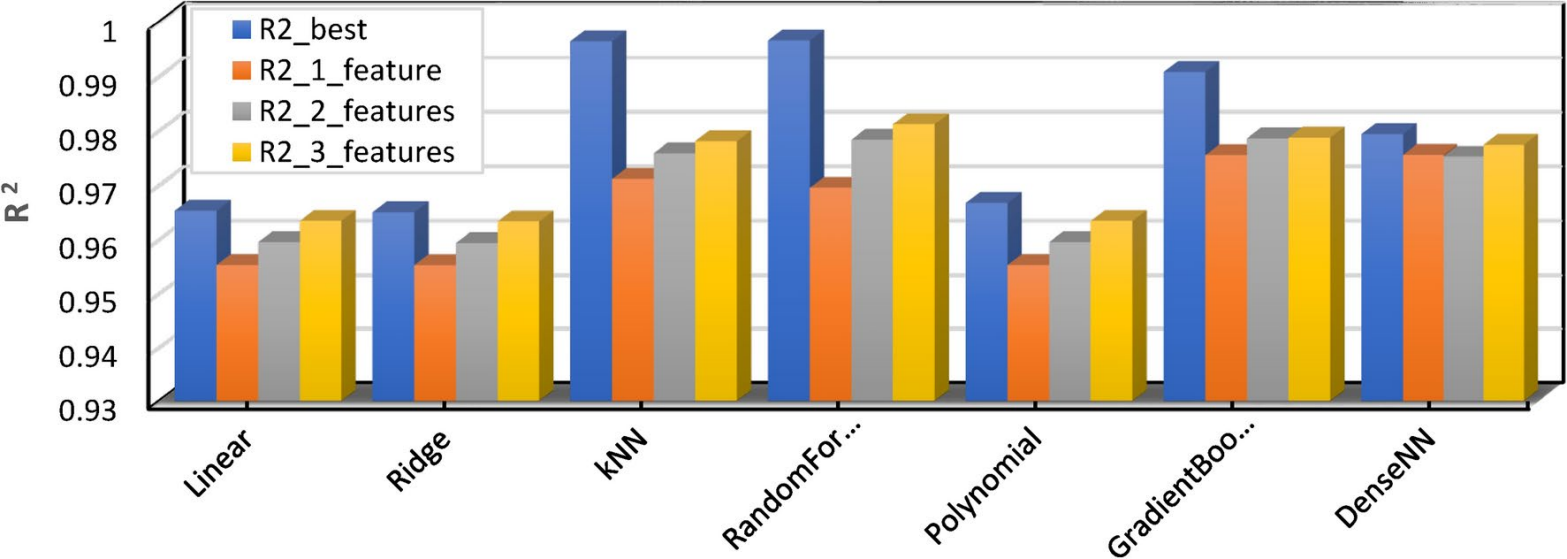
Model performance—R^2^ (coefficient of determination).

The performance of different machine learning models in predicting battery RUL was compared, including Linear Regression, Ridge Regression, k-Nearest Neighbors (kNN), Random Forest, Polynomial Regression, Gradient Boosting, and Dense Neural Network (DenseNN). Table 2 summarizes the performance metrics, namely R-squared (R^2^) for model fit and Mean Absolute Error (MAE) for prediction accuracy. Figure 15 shows the R^2^ (Coefficient of Determination) values for different machine learning models. R^2^ measures the proportion of variance in the target variable explained by the model. Higher R^2^ values indicate a better fit between the model and the data. The figure compares the R^2^ values for various features used in each model (e.g., “best”, “1_feature”, “2_features”, “3_features”). It allows for a visual comparison of how adding features affects the model’s ability to explain the data’s variability.

**Table.2.** Performance metrics summary including model fit (R^2^) and prediction accuracy (MAE).

Model	R2_best	R2_1_feature	R2_2_features	R2_3_features	MAE_best	MAE_1_feature	MAE_2_features	MAE_3_features
Linear	0.965325	0.955305	0.959547	0.963458	41.890696	42.862455	41.890696	42.709987
Ridge	0.965053	0.955281	0.959377	0.963327	41.688289	42.879856	41.688289	42.694885
kNN	0.996457	0.971175	0.975906	0.978096	6.592006	38.295498	33.720467	32.255956
RandomForest	0.996614	0.969552	0.978368	0.98121	9.230304	38.531221	32.026152	29.895593
Polynomial	0.966741	0.955305	0.959547	0.963458	41.890696	42.862455	41.890696	42.709987
GradientBoosting	0.990813	0.975557	0.9786	0.978744	21.365446	35.433984	33.701959	33.727206
DenseNN	0.97938	0.975612	0.975228	0.977441	34.988522	36.273088	36.443965	36.906502

**Fig. 16:**
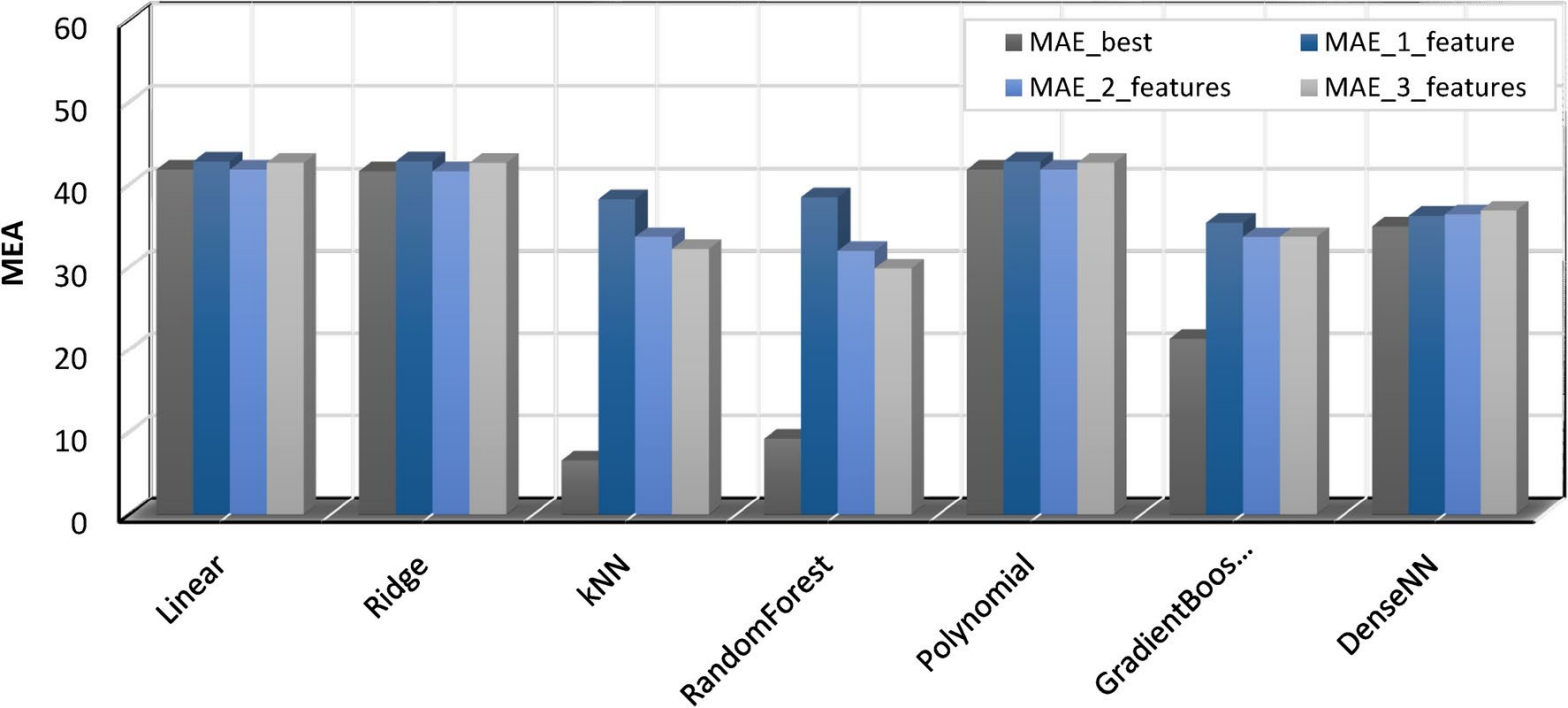
Model performance—MAE.

Figure 16 shows the MAE (Mean Absolute Error) values for different machine learning models. MAE represents the average magnitude of the difference between predicted and actual values. Lower MAE values indicate better model performance in terms of predicting accurate values. The figure compares the MAE values for different features used in each model. It allows for a visual assessment of how adding features affects the prediction accuracy of the models.

(i) **Model performance:** kNN, Random Forest, and Gradient Boosting achieved the highest R^2^ values, indicating a very good fit to the data. These models also attained the lowest MAE values, suggesting the most accurate predictions of battery RUL.
(ii) **Feature importance:** Generally, using more features (2 or 3) resulted in slightly better performance compared to using only 1 feature for most models, except Polynomial Regression which showed no change. However, the best performance wasn’t consistently achieved with all features.
(iii) **Model comparison**: kNN emerged as the leader among the tested models with the highest R^2^ and the lowest MAE, even when using only one feature. Random Forest followed closely, displaying strong performance with slightly higher MAE. Gradient Boosting also performed well but with a lower R^2^ compared to kNN and Random Forest.
(iv) **Other models**: Linear Ridge, Polynomial Regression, and DenseNN achieved good R^2^ values but lagged behind in terms of MAE, suggesting a slightly less accurate fit for predicting battery RUL.

This analysis underscores the importance of exploring different machine-learning models and feature combinations to find the optimal solution for battery RUL prediction. kNN stands out as the best performer in this specific scenario. The best R-squared value was achieved using all seven features (F1–F7) in the regression model, indicating that the full set of features provides the most comprehensive information for predicting the target variable. While simpler models with fewer features might be preferable in some cases, retaining all features seems crucial here for capturing the complex relationships influencing the outcome.

In our simulation, we conducted experiments using various feature selection techniques to identify the most relevant features for predicting the RUL of EV batteries. The resulting best_features list comprises seven sets of features, each deemed important for a specific prediction task. These sets highlight features consistently chosen by different selection methods, indicating their potential significance for prediction. Further analysis will delve into the relationships between these features and the target variable RUL to refine our understanding and potentially enhance model performance.

After an extensive comparison of multiple machine learning models, k-Nearest Neighbors (kNN) and Random Forest demonstrated the highest effectiveness for predicting RUL, especially in scenarios where a wide range of features is available. These models excel in handling complex, multi-dimensional datasets and provide accurate predictions by leveraging the relationships between various input features. However, in environments where the availability of data is limited or computational resources are restricted, Gradient Boosting stands out as a robust alternative. It offers a balance between computational efficiency and predictive accuracy, making it a versatile choice for systems that cannot support the higher resource demands of kNN and Random Forest.

As such, we recommend kNN and Random Forest for high-precision applications that require comprehensive feature analysis and can accommodate the computational overhead. Meanwhile, Gradient Boosting is more suitable for general-purpose applications where simplicity, speed, and reasonable accuracy are paramount, particularly in resource-constrained environments.

### Influence of inaccurate SOC and RUL estimation

Accurate estimation of SOC and RUL is essential for effective battery management. Inaccuracies in SOC estimation can lead to imbalanced charging, where cells are overcharged or excessively discharged, accelerating degradation and causing heat build-up that may compromise battery safety. Similarly, inaccurate RUL estimation affects battery balancing strategies, as cells closer to end-of-life may not receive targeted balancing support, leading to early failure of individual cells and reduced overall pack lifespan. Research in this area has shown the critical role of accurate SOC and RUL estimations. For example, in^46^ the authors demonstrate the use of advanced statistical techniques to improve SOC accuracy, reducing uncertainty in state estimation. Additionally, Ref.^47^ highlights the benefits of combining data-driven and physics-informed approaches to enhance RUL predictions, particularly in conditions of varying operating environments. These studies underscore the need for precise estimation methods to optimize battery life, efficiency, and safety, and support the integration of robust algorithms in our own approach to achieve these outcomes.

## Conclusion

This study presented a novel and effective active cell balancing control system for Li-ion batteries in EVs. The system leverages the average SOC as the balancing strategy and employs an inductor for energy storage. Implemented and evaluated within the MATLAB/Simulink environment, the proposed method demonstrably achieves balanced SOC across all battery cells, resulting in a substantial improvement in overall pack capacity and demonstrably extending battery lifespan. Furthermore, the study investigated the efficacy of various machine learning models for predicting the RUL of EV batteries. This investigation aimed to identify the most optimal model for precise RUL estimation and enhanced battery management strategies. Seven models were compared, including Linear Regression, Ridge Regression, k-Nearest Neighbors (kNN), Random Forest, Polynomial Regression, Gradient Boosting, and a Dense Neural Network (DenseNN). Each model was evaluated based on R-squared (R^2^) and Mean Absolute Error (MAE) metrics across different feature sets. The findings revealed kNN and Random Forest as the most proficient models, achieving the highest R^2^ values (0.996457 and 0.996614, respectively) with three features and demonstrating the lowest MAE, indicating exceptional accuracy in capturing the remaining battery life. Other models, such as Gradient Boosting, also showed promising results, suggesting potential for further exploration or hyperparameter tuning. Based on the comparison of various machine learning models, kNN and Random Forest proved to be the most effective for RUL prediction, particularly in scenarios where multiple features are available. For environments with limited data or computational resources, “Gradient Boosting” offers a robust alternative due to its balance between performance and simplicity. Thus, we recommend kNN or Random Forest for high-precision applications, while Gradient Boosting can be deployed for more general-purpose predictions. These findings highlight the practicality of employing machine learning for RUL estimation in EV batteries. Integrating these machine learning models with active cell balancing techniques empowers EV manufacturers and operators to optimize battery performance and lifespan. By accurately forecasting RUL, proactive maintenance strategies and optimized charging protocols can be implemented, leading to cost efficiencies, extended battery lifespans, and a more sustainable EV ecosystem.

## Data Availability

The datasets used and/or analysed during the current study available from the corresponding author on reasonable request.
